# Fractalkine/CX_3_CR_1_ Pathway in Neuropathic Pain: An Update

**DOI:** 10.3389/fpain.2021.684684

**Published:** 2021-07-27

**Authors:** Rita Silva, Marzia Malcangio

**Affiliations:** Wolfson Centre for Age-Related Diseases, King's College London, London, United Kingdom

**Keywords:** neuropathic pain, spinal cord, microglia, fractalkine, cathepsin S, CX_3_CR_1_

## Abstract

Injuries to the nervous system can result in a debilitating neuropathic pain state that is often resistant to treatment with available analgesics, which are commonly associated with several side-effects. Growing pre-clinical and clinical evidence over the last two decades indicates that immune cell-mediated mechanisms both in the periphery and in the Central Nervous System (CNS) play significant roles in the establishment and maintenance of neuropathic pain. Specifically, following peripheral nerve injury, microglia, which are CNS resident immune cells, respond to the activity of the first pain synapse in the dorsal horn of spinal cord and also to neuronal activity in higher centres in the brain. This microglial response leads to the production and release of several proinflammatory mediators which contribute to neuronal sensitisation under neuropathic pain states. In this review, we collect evidence demonstrating the critical role played by the Fractalkine/CX_3_CR_1_ signalling pathway in neuron-to-microglia communication in neuropathic pain states and explore how strategies that include components of this pathway offer opportunities for innovative targets for neuropathic pain.

## Introduction

Neuropathic pain is a devastating condition which affects around 7–10% of the general population globally, predominantly diagnosed in patients above 50 years of age (1). It is a chronic secondary pain condition, either as a result of peripheral (mechanical trauma, metabolic diseases, infection, etc.) or central (spinal cord injury, stroke or multiple sclerosis) nervous (somatosensory) system lesions or diseases (2). After peripheral nerve damage, both activation of sensory neurons and local inflammation occur at the site of injury. The presence of immune cells, such as macrophages (3), which release pro-inflammatory mediators and alter nociceptors' excitability (specialised sensory neurons that respond to noxious stimuli), facilitates ectopic firing and ongoing nociceptive transmission. With persistent and constant neuronal input from the periphery, dorsal horn nociceptive circuits in the spinal cord are activated, sensitised and undergo plastic changes in the CNS. Such maladaptive plasticity in the nociceptive system correlates with altered behavioural responsiveness to innocuous and noxious stimuli (4, 5). In the dorsal horn of the spinal cord, sensitisation of the first sensory synapse is characterised by a complex set of changes in synaptic efficacy, increased receptor expression and an imbalance of descending facilitatory and inhibitory modulation (6–9). Another active component to the generation of neuropathic pain involves immune cells, especially dorsal horn microglia which amplify and actively contribute to mechanisms of chronic pain (10, 11).

Data from our group and others have revealed that, after peripheral nerve damage, spinal cord microglia accumulate and proliferate in the superficial dorsal horn within the termination area of the injured peripheral nerve fibres (12–14). Peripherally injured sensory afferents instigate the change of microglial cells morphology to an activated state (15). Microglial activation is followed by the release of proinflammatory cytokines and chemokines which interact with dorsal horn neurons and modulate neurotransmission (16). As a result, these proinflammatory cytokines and chemokines are believed to contribute to increased nociceptive hypersensitivity and to the development of allodynia (response to innocuous stimuli) and hyperalgesia (enhanced response to noxious stimuli). Animal studies have spawned great interest in using glial inhibitors since blockade of microglial activity reduces nociceptive behaviours in models of neuropathic pain (14, 17, 18). Although significant spinal microgliosis is evident in both sexes of rodents after injury (19, 20), the interruption of spinal microglial activity following neuropathic injury, preferentially attenuates allodynia in male mice (19). Given the critical role of the immune system in the pathophysiology of neuropathic pain, an improved understanding of the pathways which regulate the communication between microglial and neuronal cells will shed light on innovative microglial targets for the treatment of neuropathic pain.

The identification of such bi-directional signalling pathways between CNS immunocompetent cells and neurons is critical to underpin the mechanisms underlying this interaction. Some of the major signalling pathways that mediates neuroimmune interface at the spinal cord dorsal horn level are mediated by chemokine signalling (21–23). Chemokines belong to a large superfamily of small molecules (from 8–15 kDa) and are believed to be key mediators of the interaction between neurons and neighbouring glial cells, and some chemokines exert potent chemotactic and pro-inflammatory functions. The scientific community has uncovered more than 50 chemokine ligands so far, out of which fractalkine (FKN) and its sole receptor (CX_3_CR_1_) require and deserve special attention. In this review we will examine pre-clinical evidence and focus on the role of FKN and CX_3_CR_1_ in neuron-to-microglial cell communication in neuropathic pain states and reflect on the pharmacological potential of interfering with this signalling pathway.

### FKN/CX_3_CR_1_ Pathway

FKN is a transmembrane chemokine constitutively expressed in the CNS and found in intrinsic neurons of the dorsal horn of spinal cord (24). It belongs to the CX_3_C subfamily (25) and binds to the CX_3_CR_1_ receptor. This receptor is mainly expressed by microglia in the CNS (24, 26). FKN, in its membrane-bound form, consists of an extracellular N-terminal chemokine domain, with a mucin-like stalk connecting with the cell membrane plus a transmembrane hydrophobic region and an intracellular C-terminal domain (27). FKN is also found in soluble forms, which contains the mucin stalk and the N-terminal chemokine domain, and it is released by proteolysis at a membrane-proximal region (28). This enzymatic cleavage is mediated by either the TNF-α converting enzyme (TACE, ADAM17) (28) or the metalloprotease ADAM10, which are transmembrane proteins similarly to FKN (29). FKN cleavage from neuronal membranes can also be mediated by the microglial-derived cysteine protease cathepsin S (CatS). Despite these differences in structure between full-length and soluble forms of FKN, the affinity of the chemokine for the CX_3_CR_1_ receptor is suggested to be identical (30).

A wealth of data from ours and other groups show that FKN binding to microglial CX_3_CR_1_ induces the activation of several downstream signalling pathways, especially the activation of intracellular p38 MAPK pathway that leads to the release of CatS and IL-1β (26, 31). The activation of this pathway is linked to nociceptive facilitation after nerve injury (18, 32–34). Similar outcome has been reported in bone cancer pain models which have a neuropathic pain component (35). Pain development in this model correlates with an increased dorsal horn microgliosis and increased expression of p-p38 in microglia (36). Indeed, this chemokine pair FKN/CX_3_CR_1_ is involved in neuropathic pain development and maintenance via neuron-microglia interaction in the dorsal horn, and upregulation of CX_3_CR_1_ expression is observed when microgliosis is present (21, 37–40). Despite differences observed, sexual dimorphism in FKN/CX_3_CR_1_ pathway in the spinal cord is yet to be established.

### FKN/CX_3_CR_1_ Pathway and Pain

Accumulating evidence over the last fifteen years suggest an important role of microglia in the pathogenesis of neuropathic pain (41, 42). Following peripheral nerve injury, upregulation of CX_3_CR_1_ (24) is observed in spinal microglia in association with marked mechanical allodynia (43). Thermal hyperalgesia and mechanical allodynia can also be elicited in naïve animals by an intrathecal injection of FKN (44) and both effects are abrogated in CX_3_CR_1_ knockout mice (34, 45). In addition, the administration of a neutralizing antibody against CX_3_CR_1_ (46, 47) reduces pain-like behaviours in neuropathic pain models, indicating that microglia-mediated mechanisms contribute to nociceptive hypersensitivity. Injection of FKN, after unique binding to CX_3_CR_1_, activates p38 MAPK signalling pathway (34). Selective inhibition of p38-MAPK with skepinone or SB20358 through intrathecal delivery reduced mechanical allodynia in male rodent models of neuropathic pain (48) highlighting the role of phosphorylated p38 MAPK in neuropathic pain. In addition, a study published by Bäckryd and co-workers has found that FKN and CatS levels are higher in the cerebrospinal fluid (CSF) of fibromyalgia patients when compared to healthy individuals (49).

Besides a well-established role in neuropathic pain at spinal cord level and increased FKN in the CSF, some reports have elucidated the FKN/CX_3_CR_1_ role at a supraspinal level. In the brain, increased microglial expression has been reported in pain-related areas such as the thalamus (50) or the periaqueductal grey area (PAG) (51). In a recent study, an upregulation of *CatS, CX*_3_*CR*_1_ and *FKN* mRNA and CX_3_CR_1_ protein expression was observed in the ventral posterolateral thalamic nucleus after spinal nerve ligation (SNL) in rodent models (52). This is further supported by a study examining patients suffering from lumbar chronic pain in which evidence for microglial activation in the thalamus is noticed (53). Evidence also shows that intracerebroventricular administration of FKN causes thermal hyperalgesia in rodents and is accompanied by an increase in p38 MAPK phosphorylation (54).

These data provide a better understanding of the pathophysiological processes in the spinal cord and in the brain highlighting the potential of the FKN/CX_3_CR_1_ system as a target for the treatment of neuropathic pain.

### CatS/FKN/CX_3_CR_1_ Pathway and Pain

CatS is a lysosomal enzyme belonging to the papain family of cysteine proteases (55) preferentially expressed in mononuclear phagocytic cells (56). CatS expression has been observed in dendritic cells, B cells, macrophages and microglia, which act as antigen presenting cells (APCs) (57). The activity of CatS is not restricted to intracellular compartments since the release of enzymatically active protease has been observed in a number of cell types, including macrophages and microglia (26). Due to its expression in immune cells and the direct involvement of CatS in antigen presentation, this enzyme has been linked to several autoimmune conditions such as multiple sclerosis (58) and rheumatoid arthritis (59, 60).

Like most cathepsins, CatS is a small and monomeric endopeptidase (61). It is synthesized as an inactive zymogen in the lysosomal compartment (62). After removal of the pro-peptide by other proteases, CatS becomes enzymatically active (63). This protease plays an important role in adaptive immune responses by regulating MHC class II surface expression and by cleaving the invariant chain p10 (Lip10)—a fragment of the MHC class II-associated invariant chain peptide (64). Mice lacking the *Ctss* gene display diminished MHC class II (MHCII) antigen presentation (65). In comparison to many other cysteine cathepsin family members, CatS tissue expression is very restricted. Biochemically, this endopeptidase has the ability to retain activity at a neutral pH and this property showcases its increased potential to be involved in extracellular proteolytic activities (63).

**Figure 1:**
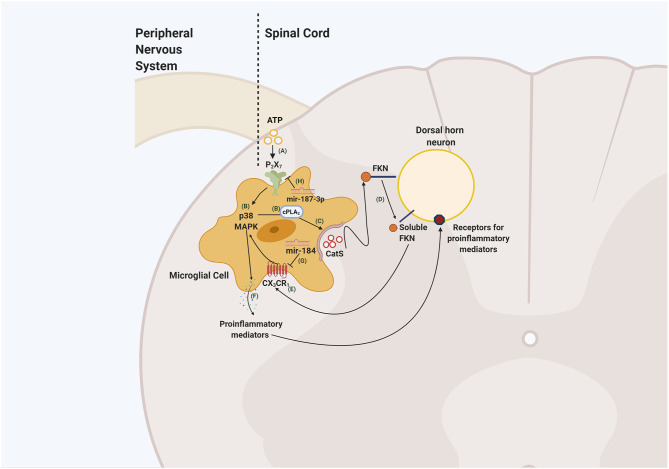
Schematic illustrating neuron-to-glia interaction through the CatS/FKN/CX_3_CR_1_ pathway in the spinal cord dorsal horn. ATP released by damaged primary afferents and dorsal horn neurons **(A)** lead to the activation of P_2_X_7_ receptors. The activation of these receptors phosphorylates p38 MAPK signalling pathway and phospholipase A2 (cPLA_2_) **(B)** resulting in the release of CatS **(C)**. CatS cleaves FKN present in the membrane of the spinal cord dorsal horn neuron **(D)**. The soluble form of FKN interacts with the CX_3_CR_1_ receptor located in microglia **(E)** that phosphorylates p38 MAPK, induces the release of proinflammatory mediators **(F)** that may sensitise spinal cord dorsal neurons and contribute to the development of central sensitisation and neuropathic pain. Mediating the expression of mir-184 inhibits the activation of CX_3_CR_1_ **(G)** consequently reducing the release of proinflammatory mediators and microglial activation. On the other hand, upregulating mir-187-3p **(H)** through the administration of its mimic, downregulates P_2_X_7_ expression, potentially disrupting the liberation of CatS. This figure was created with BioRender.com.

In addition to the intracellular function, CatS also shows extracellular activity when it is released by macrophages and microglia. As reported by Clark and co-workers, upon release of CatS, the latter interacts with FKN on neurons, cleaving into its soluble form that further binds to the CX_3_CR_1_ receptor located on microglia. The activation of this receptor leads to the phosphorylation of p38 MAPK pathway contributing to the release of proinflammatory cytokines, such as IL-1β to the extracellular environment (66). These can activate neighbouring neurons and contribute to increased neuronal excitability (67, 68). However, the CatS/FKN/CX_3_CR_1_ signalling pathway is only fully operational in the presence of high concentrations of adenosine tri-phosphate (ATP) which contributes to the activation of the P_2_X_7_ receptor (69), reflecting the critical role of ATP to induce the release of CatS (Figure 1).

CatS expression is upregulated after peripheral nerve injury in the spinal cord dorsal horn and is accompanied by an increase in mechanical sensitivity. This is abrogated by the administration of a neutralizing antibody against FKN suggesting that CatS requires CX_3_CR_1_ to exert pro-nociceptive activity (34). This suggestion is further validated by the observation that CatS intrathecally injected in CX_3_CR_1_ knockout mice fails to induced and mechanical allodynia (34). In addition, a recent study has shown that one day post peripheral nerve injury CatS mRNA levels are upregulated in the ipsilateral side of the spinal cord and similar observations could be verified after intrathecal administration of colony-stimulating factor 1 (CSF1) (70). This interaction between CSF1 and CatS sheds light into new players that contribute to microglial activation and CatS release. Notably, this further reinforces the idea that CatS is a pro-nociceptive contributor for the central mechanisms underlying neuropathic pain. Despite playing an undeniable pro-nociceptive role centrally, it is important to note that CatS also exerts effects in the periphery by acting on targets such as the protease-activated receptor 2 (PAR_2_) (71). Activation of this receptor by CatS through enzymatic cleavage has been shown to contribute to preclinical pain (72) and reflects the versatile nature of CatS, emphasizing its powerful potential as a therapeutic target.

### Regulation of CatS/FKN/CX_3_CR_1_ Pathway Through miRNAs

Several studies have demonstrated that noncoding RNAs, especially microRNAs, are altered in pain-related regions and these changes are linked with neuropathic pain pathology (73, 74). Several microRNAs have been linked to chemokine signalling. For instance, miR-23a is downregulated in the spinal cord after nerve injury and an increase of the expression of this noncoding RNAs reduces CXCR_4_ expression and attenuates pain-like behaviours (75). More recently, in a model of bone cancer pain (BCP), the expression of *cx3cr1* mRNA expression was upregulated along with increased microglial activation in the spinal cord. Computational analysis revealed that *cx3cr1* is a target gene for miR-184 and by activating miR-184, microglial CX_3_CR_1_ expression is downregulated (76). Furthermore, in models of ischemia-reperfusion (IR)-induced pain hypersensitivity, downregulation of P_2_X_7_ receptor expression by an intrathecal injection of the mimic-187-3p was associated with reduced pain hypersensitivity as well as reduction in cleaved caspase-1 and IL-1β protein levels in the spinal cord (77). By blocking, inactivating, or reducing the expression of P_2_X_7_ receptors, our prediction is that CatS release by microglial cells would be halted and, consequently, FKN would not be cleaved into its soluble form (Figure 1). Therefore, miRNAs constitute an innovative and effective strategy to target several players within a pathway involved in neuropathic pain mechanisms.

### CatS/FKN/CX_3_CR_1_ Pathway: Therapeutic Approaches and Limitations

Based on the emerging appreciation for the role of P2X receptors in mediating nociceptive neurotransmission, several P2X receptors have advanced into clinical trials for inflammation and pain. For instance, intraperitoneal administration of A-438079, a selective competitive P_2_X_7_ antagonist and CNS penetrant compound (78), reduces pain-like behaviours in three different rodent animal models of neuropathic pain (79). Regardless of all the promising results obtained in the pre-clinical setting, most of the P_2_X_7_ receptor antagonists have not been approved for pain management until today (80).

On the other hand, several pre-clinical studies have reported a successful attenuation of allodynia and hypersensitivity by intrathecal administration of the non-selective CatS inhibitor LHVS as well as following administration of MIV-247, an orally available selective CatS inhibitor that can penetrate the CNS (34, 81). Several other CatS inhibitors (VBY-036 and VBY-891) have gone through Phase I clinical trials and were considered safe for further efficacy studies. Furthermore, the development of a CatS/CatK inhibitor (SAR113137) entered clinical trials for pain management but it was later halted due to initial safety setbacks (82). However successful in preclinical experiments, none of these inhibitors have yet gone through Phase II clinical trials.

At present, molecules targeting P_2_X_7_ receptors and CatS have not progressed in clinical trials. However, the CX_3_CR_1_ inhibitor AZD8797, which has shown efficacy in models of multiple sclerosis resulting in reduced paralysis, is a good candidate to be considered for treatment of neuropathic pain (83) and its' use for the management of pain may be considered. However, the active involvement of this chemokine pair in other conditions besides chronic pain indicates that pharmacological tools that alter CX_3_CR_1_ signalling may result in side effects. For instance, whilst the activation of FKN and/or CX_3_CR_1_ signalling may provide novel opportunities for the treatment of Alzheimer's Disease (AD) (84), this does not represent a strategy for neuropathic pain where a blockade of FKN and/or CX_3_CR_1_ would be desirable. Even though neurogenerative and chronic pain conditions are both associated with neuroinflammation, including microglial activation, the specific role of the FKN/CX_3_CR_1_ signalling pathway in each situation may differ and thus remain to be investigated.

### CatS/FKN/CX_3_CR_1_ Pathway: Future Avenues

Despite great progress in the study of chemokines and their involvement in the development of pain, especially regarding the FKN/CX_3_CR_1_ pair, very few analgesic drugs targeting chemokines have reached later phases of clinical trials. Molecules that target soluble FKN, which is known to mediate nociception, or its respective signalling, may provide reduced side and stronger analgesic effects. Furthermore, targeting upstream regulators of FKN transcription, such as *Stat3* (signal transducer and activator of transcription 3) could be explored as a new avenue to regulate FKN expression in neurons.

Considering the paucity of therapies for the treatment of neuropathic pain, we suggest that future studies could investigate the role of CatS/FKN/CX_3_CR_1_ in supraspinal areas which may complement research conducted in the spinal cord and in the dorsal root ganglion (DRG). Currently, little information is provided regarding the effect of this signalling pathway in supraspinal areas in a neuropathic pain context. Uncovering the role of FKN and CX_3_CR_1_ in the pain-related areas in the brain under neuropathic pain states may aid in the development of innovative therapeutic approaches.

## Conclusion

This chemokine system plays an important role in the development of neuropathic pain in preclinical studies. The identification of these neuron-microglia interactions during neuropathic pain states has led to the identification of microglial targets such as the chemokine receptor CX_3_CR_1_, the lysosomal protease CatS and the P_2_X_7_ receptor. The inhibition or downregulation of these microglial targets, by using different therapeutic tools (inhibitors, miRNAs, etc.) still constitute a powerful tool for addressing whether modulation of this signalling pathway can attenuate neuropathic pain.

## Author Contributions

All authors contributed to manuscript revision, read, and approved the submitted version.

## Conflict of Interest

The authors declare that the research was conducted in the absence of any commercial or financial relationships that could be construed as a potential conflict of interest.

## Publisher's Note

All claims expressed in this article are solely those of the authors and do not necessarily represent those of their affiliated organizations, or those of the publisher, the editors and the reviewers. Any product that may be evaluated in this article, or claim that may be made by its manufacturer, is not guaranteed or endorsed by the publisher.
